# The Impact of a Single Session of Sprint Interval Training (SIT) on Pain Pressure Threshold (PPT) in Healthy Adults

**DOI:** 10.3390/sports14060237

**Published:** 2026-06-08

**Authors:** Francesco Pacenza, Gary Brickley, Umberto Crainich, Francesco Bettariga, Anthony Turner, Luca Maestroni

**Affiliations:** 1 School of Sport and Health Sciences, University of Brighton, Brighton BN1 9PH, UK; pacenza.francesco@gmail.com (F.P.); g.brickley@brighton.ac.uk (G.B.); 2 Move Physiotherapy, Via Mazzini 12/A, 33170 Pordenone, Italy; umberto.crainich@gmail.com; 3 London Sports Institute, Middlesex University, Stone X Stadium, Greenlands Lane, London NW4 4BT, UK; 4 School of Human Performance, Rehabilitation and Population Health, University of Technology Sydney (UTS), Moore Park, NSW 2021, Australia; francesco.bettariga@uts.edu.au; 5 Exercise Medicine Research Institute, School of Medical and Health Sciences, Edith Cowan University, Joondalup, WA 6027, Australia

**Keywords:** hypoalgesia, exercise, interval training

## Abstract

Different forms of aerobic exercise lead to Exercise-Induced Hypoalgesia (EIH). The aim of this study was to investigate the acute effect of a single session of Sprint Interval Training (SIT) on EIH. A total of 44 recreationally active young adults participated in a single SIT session on a cycle ergometer, consisting of a 2 min warm-up, 3 × 20 s “all-out” cycling bouts interspersed with 2 min of active recovery, and a 3 min cool-down. Pain Pressure Threshold (PPT) measurements were taken on the quadriceps (working muscle) and thenar eminence (resting muscle) before and after the session. Additionally, average power generated during the sprints and the Rating of Perceived Exertion (RPE) at the end of the session were recorded. Percentage changes from baseline to post-session were calculated for each participant and averaged. Paired-samples *t*-tests assessed pre–post differences in both local and distal PPT, with Cohen’s *d* effect sizes (ES) and 95% confidence intervals (CIs) determining the magnitude of differences. Pearson correlation examined the relationship between average power, RPE, and changes in local and distal PPT. All results were statistically significant (*p* < 0.001). A single SIT session induced a large hypoalgesic response at both the local site (*d* = 1.79; 95% CI 1.31–2.27) and the distal site (*d* = 1.16; 95% CI 0.77–1.54). No significant correlations were found between average power, RPE, and local and distal PPT changes. In this exploratory single-arm study, PPT increased significantly at both local and distal sites following a single 10 min SIT session, consistent with an acute EIH response; however, in the absence of a control condition, these findings should be interpreted with caution.

## 1. Introduction

The World Health Organization (WHO) defines physical activity as any bodily movement produced by skeletal muscles that requires energy expenditure [1]. In detail, the WHO guidelines emphasize the importance of incorporating both aerobic and resistance training into routines to prevent the possible onset of non-communicable diseases, including cardiovascular diseases, chronic respiratory diseases, type 2 diabetes mellitus, and cancer [1]. At least 150–300 min of moderate-intensity or 75–150 min of vigorous-intensity aerobic physical activity throughout the week and strengthening exercises at moderate or high intensity involving all major muscle groups at least twice per week are recommended in adults. Exercise, which is a sub-category of physical activity, is planned, structured, and repetitive, aiming to improve or maintain health.

A growing body of evidence suggests that regularly performing brief vigorous exercise not only is efficacious in improving aerobic performance, but also in reducing the risk of cardiometabolic diseases [2]. This includes intermittent bouts performed above moderate intensity (>64% of maximal oxygen uptake [⩒O2_max_]) interspersed with recovery periods of light work or rest, commonly known as high intensity interval training (HIIT) [3,4]. Particularly, a HIIT modality involving “all-out” or “supramaximal” efforts (>100% of ⩒O2_max_) is referred to as sprint interval training (SIT) [5]. To date, one of the most adopted SIT protocols involves 4–6 bouts of “all-out” 30 s cycling sprints with 4 min recovery intervals between sprints [6]. However, recent evidence suggests that sprints as short as 10 s can elicit similar benefits, highlighting the need for future investigations to explore the differences among various training protocols [4,7]. Among low-volume SIT models, protocols based on 3 × 20 s all-out cycling bouts have received particular attention because they can induce relevant physiological adaptations despite a markedly reduced exercise volume and total time commitment. Previous studies have shown that this protocol can improve skeletal muscle oxidative capacity and cardiometabolic markers in overweight/obese adults, and can induce adaptations comparable to moderate-intensity continuous training despite substantially lower exercise volume and time commitment [8,9,10].

Exercise has been shown to positively affect musculoskeletal pain [11]. Indeed, recommendations from high-quality clinical practice guidelines for musculoskeletal pain include exercise as first-line treatment regardless of the anatomical region affected [12,13]. Beyond the specific benefits on cardiovascular, bone, cartilage, muscle, and tendon properties induced by resistance and aerobic training [14,15,16], it has been documented that as little as a single exercise session can reduce pain sensitivity in healthy individuals and clinical populations [17]. This acute effect is underpinned by a phenomenon known as exercise-induced hypoalgesia (EIH), which arises from the activation of various central mechanisms, including opioid, endocannabinoid, and serotonergic pathways [18]. Specifically, EIH is thought to result from the activation of descending pain inhibitory pathways by releasing endogenous opioid-related substances at central nervous system sites and locally at the trained muscle areas, as well as by enhancing serotonergic and endocannabinoid activity, which modulate nociceptive processing regions in the brain and spinal cord [19,20]. The acute reduction in pain sensitivity following a single session highlights the therapeutic potential of exercise as an intervention in clinical settings and as a self-management strategy. EIH is commonly measured using the pain pressure threshold (PPT) before and after the exercise session [17,21], with an increase in PPT being indicative of EIH [22]. EIH leads to a significant reduction in pain sensitivity both locally (i.e., in the body region being exercised) and, to a lesser extent, distally (i.e., in a remote body region) [17,23]. Research has shown that practicing intense physical training leads to increased baseline pain tolerance and more efficient pain modulation [19,24], although details about participants’ exposure to training are not commonly reported in EIH studies. Overall, resistance exercise (RE) and aerobic exercise (AE) have consistently been shown to induce EIH in healthy adults and, with less predictability, in musculoskeletal pain conditions [17]. A recent systematic review highlighted that a single session of AE, ranging from 30 s to 30 min, induced large EIH (g = 0.85 [95% CI, 0.13 to 1.58]) in healthy individuals when compared to rest or sham exercise [22]. Preliminary research has also indicated that a higher intensity (70% of heart rate reserve) during a vigorous aerobic session can induce an even larger hypoalgesic effect (*d* = 1.07) compared to a moderate intensity session in healthy adults (*d* = 0.24) [25]. This trend was observed in studies investigating different exercise durations, ranging from 30 min [26,27] to longer exercise durations of up to 60 min [28]. Nonetheless, limited evidence is available regarding the effect of short repeated “all-out” bouts, characteristic of SIT, on EIH in both healthy and clinical populations. The available studies have shown contrasting findings after a single all-out bout [29], repeated maximal sprint cycling exercise [30], or interval-based exercise protocols with heterogeneous bout durations [31]. However, it remains unclear whether this specific time-efficient 3 × 20 s SIT model, previously investigated for cardiometabolic and skeletal muscle adaptations [8,10], can acutely modulate pain sensitivity. Therefore, the present study extends this established SIT protocol to the assessment of EIH by evaluating both local and distal PPT responses in healthy adults. Recent controlled evidence further suggests that very short all-out ergometer-based exercise can induce EIH in healthy trained individuals, supporting the need to investigate brief repeated SIT protocols and their effects on both local and distal PPT responses [20,32]. Considering the paucity of studies investigating EIH following a single SIT session, it appears of utmost importance to clearly elucidate the magnitude of EIH in healthy individuals. Furthermore, it remains unclear whether changes in PPT are associated with different training parameters (i.e., power during sprints and rate of perceived exertion [RPE]). Taken together, considering the proven health benefits, minimal time commitment, and practical applicability of SIT [3,33], we therefore aimed to (1) investigate the acute EIH effect of a single session of SIT in healthy adults; and (2) examine the relationship between average power during sprints, RPE, and local and distal PPT changes. We hypothesized that a single SIT session would induce a significant increase in PPT, corroborating the EIH effect, and this would be related to sprint intensity and perceived effort.

## 2. Materials and Methods

### 2.1. Subjects

Healthy adults volunteered to take part in the study (Table 1). Sample size was established through an a priori power analysis using G*Power (Version 3.1, University of Düsseldorf, Düsseldorf, Germany), based on a two-tailed paired-samples *t*-test, an alpha level of 0.05, statistical power of 0.90, and a moderate effect size of *d* = 0.50, consistent with conventional effect size interpretation [34]. This indicated that 44 participants were required. Eligible participants were included if they fulfilled the following inclusion criteria: (1) ≥18 and ≤40 years of age; (2) regularly engaged in recreational physical activity (International Physical Activity Questionnaire score moderate to high); (3) had medical clearance for strenuous physical activity; (4) had no history of musculoskeletal pain in the 6 months prior to the experiment; (5) had no prior orthopaedic surgeries performed in proximity to the wrist or femur in the last 12 months; and (6) had no cardiovascular, metabolic, neurological, or cognitive diseases. All subjects were informed about the purpose of the study and gave informed consent before the start of the experimental procedures, according to the Declaration of Helsinki 2013. Ethical approval was granted by the University of Brighton School of Sport & Health Science Research Ethics Committee (2023-12022).

### 2.2. Materials

A stationary cycle ergometer (BikeErg, Concept2, Inc., Morrisville, VT, USA) was used for the experiment. Sprint intervals were programmed using proprietary software (ErgData by Concept2, v2.2.17), which recorded the average power of each interval. A heart rate (HR) monitor (Polar H10, Polar Electro Oy, Kempele, Finland) with a chest strap was connected to the participant to monitor HR throughout the entire session. PPT was assessed using a digital algometer (FPX-25, Wagner Instruments, Greenwich, CT, USA) equipped with a 1 cm^2^ flat, rounded rubber tip. Overall RPE was assessed at the end of the session with the Borg CR10 scale [35].

### 2.3. Study Procedure

Participants were instructed not to engage in any physical activity on the day of the experiment and to refrain from consuming heavy meals or alcohol prior to the study. During the initial assessment, PPT was measured at two distinct body areas. The first measurement targeted the primary muscle group involved (i.e., quadriceps muscle), referred to as the local site. The second measurement was conducted on the thenar eminence, a remote and non-exercised body part referred to as the distal site. Measurements were taken three times on both the quadriceps (PPT Thigh) and thenar eminence muscles (PPT Hand). PPT assessments were always performed in the same fixed order, with the quadriceps assessed first, followed by the thenar eminence. Each measurement was separated by a 30 s rest interval, following the recommendation by Liew et al. [36]. These assessments aimed to determine the reliability of PPT measurements at baseline. PPT assessments for the quadriceps muscle were taken at a point located midway between the superior pole of the patella and the anterior superior iliac spine [21,37]. PPT measurements for the thenar eminence muscles were obtained at the midpoint between the head and the base of the first metacarpal, with the hand resting on the thigh [38]. To ensure consistency across baseline testing and subsequent exercise phases, both measurement points were marked with a washable skin marker. A consistent pressure was applied perpendicular to the marked point until the subjects reported the initial onset of discomfort or painful sensation [39]. Participants were instructed to keep their eyes closed and were not allowed to view the algometer monitor display. PPT measurements were conducted by a trained clinician who also could not see the data displayed on the monitor. The results were recorded, and the mean scores were used for subsequent analysis.

The stationary bike built-in software (ErgData by Concept2) was utilized to program the workout, specifying the number and duration of each interval and recording the average power of each sprint, which was subject to secondary analysis. Participants were then asked to sit on the stationary bike, and the saddle height and handlebar position were individually adjusted for maximum comfort. Following these adjustments, a familiarization phase was conducted during which participants pedaled against varying resistances determined by the BikeErg damper setting. The damper setting was self-selected by each participant to identify the resistance perceived as most suitable to facilitate maximal power output during the sprint phases [8]. Once selected, the damper setting was kept constant throughout the SIT session for each participant. After completing the familiarization phase, participants rested for 5 min and subsequently underwent a structured SIT session [9,10,40,41]. The session commenced with a 2 min light cycling warm-up. Following the warm-up, three sets of 20 s all-out sprint efforts were performed, with each set separated by a 2 min interval of relative rest involving light cycling. Subsequently, a 3 min light cycling cool-down phase was performed. Throughout the warm-up, rest intervals, and cool-down phases, participants were instructed to maintain their effort at a consistent 50 W, as indicated on the bike monitor. PPT was measured at both local (quadriceps muscle) and distal (thenar eminence) sites immediately after completion of the SIT protocol, following the 3 min cool-down phase, using the same fixed order as baseline assessment. Additionally, subjects were asked to rate their overall RPE on a CR10 Borg scale [35].

### 2.4. Statistical Analysis

All data were initially recorded as mean and standard deviation (SD) in Microsoft Excel^®^ 2010 and later transferred to SPSS (version 25.0; SPSS, Inc., Armonk, NY, USA). Prior to transfer to SPSS, all data were reviewed through visual inspection and range checks to identify potential data entry errors and detect implausible values. Normality was analyzed using the Shapiro–Wilk test, with *p* > 0.05 indicating that data were normally distributed. An average-measures two-way random intraclass correlation coefficient (ICC) with absolute agreement and 95% confidence intervals, and coefficient of variation (CV), were used to assess the within-session reliability of test measures at baseline. Intraclass correlation coefficient values were interpreted as follows: >0.9 excellent, 0.75–0.9 good, 0.5–0.75 moderate, and <0.5 poor [42]. The CV was calculated using the formula: (SD [trials 1–3]/average [trials 1–3] × 100), with values of ≤10% deemed acceptable [43]. The standard error of measurement (SEM) was calculated using the formula SEM = SD × SQRT (1 − ICC), providing a quantitative measure of the precision of individual scores. Paired-samples *t*-tests were used to examine differences in PPT locally and distally following the protocol. Cohen’s *d* effect sizes (ES) with 95% confidence intervals were calculated to interpret the magnitude of these differences, with the following classification: 0.2, 0.5, and 0.8 for small, moderate, and large effect sizes, respectively [34,44]. Significance was set at *p* < 0.05. As secondary analysis, Pearson correlation was used to assess the association between average power expressed during the sprint intervals, RPE, and PPT changes at local and distal sites. Ninety-five percent confidence intervals for Pearson correlation coefficients were also reported.

## 3. Results

Forty-four participants were included in the final analysis (Table 1). Data were normally distributed. PPT Thigh and PPT Hand showed excellent reliability scores, as documented by CV ≤ 10% (PPT Thigh = 4.83%; PPT Hand = 6.55%) and high ICC values (PPT Thigh = 0.97, 95% CI 0.95–0.98; PPT Hand = 0.96, 95% CI 0.93–0.98). The SEM was 0.22 for PPT Thigh and 0.25 for PPT Hand.

**Figure 1:**
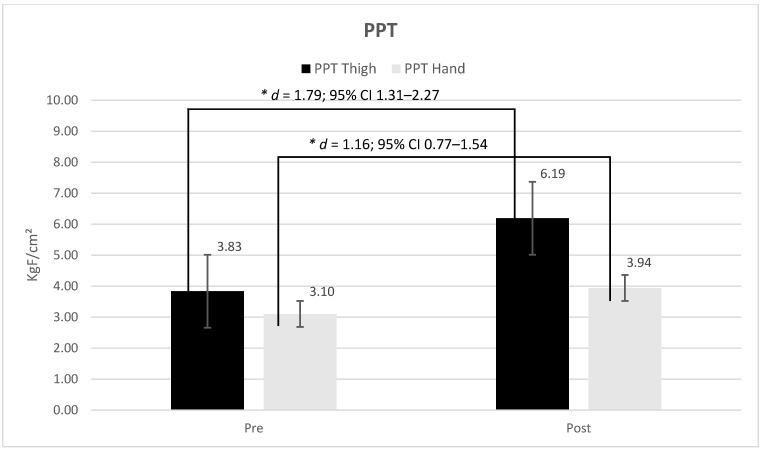
Pre–post intervention changes on Pain Pressure Threshold (PPT) for local site (PPT Thigh) and distal site (PPT Hand). *: statistical significant difference between pre- and post-intervention measurements (*p* < 0.05).

Following the SIT session, large significant increases in PPT were observed both locally (PPT Thigh) (*d* = 1.79, 95% CI 1.31–2.27; *p* < 0.001) and distally (PPT Hand) (*d* = 1.16, 95% CI 0.77–1.54; *p* < 0.001). Percentage change from baseline was 70.9% ± 56.16% for PPT Thigh and 32.84% ± 31.75% for PPT Hand (Table 2, Figure 1). No significant correlations (*p* > 0.05) were found between RPE (7.8 ± 0.9) or average power achieved during the sprint intervals (543 ± 111 W) and changes in PPT Thigh and PPT Hand (Table 3).

## 4. Discussion

The aims of this exploratory single-arm pre–post study were twofold: (1) to investigate acute PPT changes following a single brief SIT session in healthy adults, and (2) to examine the relationship between total average power during sprints, RPE, and changes in local and distal PPT. Our results showed that PPT increased significantly at both the local and distal sites after the SIT protocol, with increases of 70.9% (*d* = 1.79, 95% CI 1.31–2.27; *p* < 0.001) and 32.8% (*d* = 1.16, 95% CI 0.77–1.54; *p* < 0.001), respectively. These findings are consistent with an acute hypoalgesic response following SIT, as PPT at both the local and distal sites demonstrated excellent reliability at baseline. However, because no non-exercise or time-matched control condition was included, causal attribution of the observed changes solely to the SIT protocol should be made with caution. Contrary to our hypothesis, no significant correlations were found between RPE or sprint power and changes in local and distal PPT.

Notably, the EIH magnitude observed in our study was greater than that reported in the comparable literature [29,30,31]. This may be related to the exercise intensity used in our protocol, characterized by shorter rest intervals (i.e., 2 min) compared to previous studies (i.e., 3 min) [31], and by multiple all-out bouts compared to single bouts [29]. To contextualize these changes, Vaegter et al. used an increase greater than 1 × SEM to classify participants as EIH responders, reporting SEM values of 43–57 kPa for quadriceps PPT and 19–27 kPa for trapezius PPT [36]. In the present study, the absolute mean increases were 2.36 kgf/cm^2^ for PPT Thigh and 0.84 kgf/cm^2^ for PPT Hand, corresponding approximately to 231 kPa and 82 kPa, respectively. Thus, the local PPT increase clearly exceeded previously reported SEM values for quadriceps PPT, whereas the distal response should be interpreted more cautiously because the comparison is based on a different remote site. In our study, high intensity was encouraged through pre-explanation and verbal encouragement during each sprint to ensure that maximal effort was achieved. This was reflected by high perceived exertion (RPE = 7.8 ± 0.9) and high average 20 s sprint power (543 ± 111 W), consistent with SIT protocols [3]. Existing literature indicates that higher aerobic intensities (>75% ⩒O2_max_) can foster greater EIH [22], which is in line with our findings, especially when exceeding 200 W [44]. However, our secondary analysis revealed no correlation between RPE or sprint power output and EIH, suggesting that the individual changes in PPT observed in our sample were not associated with these variables within the consistently high-intensity stimulus selected for our protocol. This finding is consistent with a recent study [29], which observed that a single 30 s all-out bout induced a small hypoalgesic effect without significantly correlating with blood lactate levels or muscular pain, both parameters related to exercise intensity [45]. This absence of significant correlations should also be interpreted in light of a likely restriction of range. Because all participants performed maximal efforts and showed consistently high average power output and RPE values, the limited variability in these exercise intensity markers may have reduced the ability to detect linear associations with PPT changes. However, the underlying reasons are not fully understood, and further research is required to examine which training parameters may influence EIH. It is known that acute exercise is a form of physical stress that increases hypothalamic–pituitary–adrenal axis activation, with consequent release of various neurotransmitters (e.g., opioids, endocannabinoids, serotonin, dopamine) primarily attributed to the reduction in pain sensitivity [18,46]. Recent research suggests that catecholamine (i.e., adrenaline, noradrenaline) production levels may enhance the EIH effect, owing to their production in the central nervous system and in proximity to the contracting muscles, which may explain the greater EIH documented at the local body region in our cohort [20,47]. Although speculative, it could be hypothesized that when multiple all-out bouts are used, the anaerobic energy system is greatly stimulated [48], with preliminary findings suggesting that surpassing the anaerobic threshold could lead to a greater hypoalgesic effect [28]. Indeed, it has been documented in incremental cycling ergometer tests, particularly for workloads exceeding 300 W, as in our study, that there is an increased release of β-endorphin [49,50], a morphine-like neuropeptide involved in peripheral pain modulation [51]. However, because physiological biomarkers such as lactate, β-endorphin, endocannabinoids, catecholamines, or heart rate variability were not measured, these mechanistic interpretations remain speculative and should be considered hypothesis-generating. This may represent an attractive field for future investigations aiming to explore the benefits of EIH in musculoskeletal pain and the measurable phenotypic characteristics that are most predictive of individual variation in EIH.

It should be acknowledged that EIH is a complex phenomenon, involving several interconnected biological mechanisms such as the interaction between the opioid, endocannabinoid, and serotonergic systems [18]. Additionally, psychosocial factors such as fear of pain, pain catastrophizing, and beliefs about the perceived threat of exercise may contribute to its clinical manifestation in musculoskeletal pain [18,52]. A recent systematic review demonstrated that AE performed at submaximal intensity (50–75% ⩒O2_max_) reduces pain sensitization (median % improvement by 10.6%) in individuals with low back, neck, shoulder, elbow, and knee pain [53], whereas AE at low or self-selected intensity did not result in either local or distal EIH in patients suffering from persistent pain [54]. Interestingly, to date, no studies have investigated the EIH magnitude induced by SIT protocols in clinical populations.

Taken together, beyond the well-known benefits of SIT in improving cardiorespiratory capacity [9,10], its wide applicability outside the laboratory environment [33], its low time commitment [7], and its enjoyability compared to longer types of training [55], SIT may represent an efficient method to induce EIH in healthy individuals. From a rehabilitation perspective, brief SIT protocols may warrant consideration as time-efficient adjuncts to exercise-based programs for musculoskeletal conditions, such as tendinopathies in athletic populations, particularly when maintaining cardiorespiratory fitness and facilitating acute pain modulation are clinically relevant objectives. However, these applications remain hypothetical, as the present study included only healthy adults and did not assess clinical outcomes in people with musculoskeletal pain. A notable strength of our study lies in its clinical applicability, as a substantial hypoalgesic effect was observed both locally and distally with minimal time commitment and no specialized equipment. Moreover, our analysis revealed excellent reliability at baseline, as shown by our CV and ICC scores, which strengthens confidence in the reported results. Future studies could assess PPT changes at multiple post-exercise time points, for example, at 30 and 60 min, to determine the time course and persistence of the observed PPT response. Our data were limited to healthy adults regularly engaged in moderate-to-high recreational physical activity, a population likely to show higher EIH responses [18,24] than clinical populations [17] or low-to-moderately active individuals [19]. Although participants were classified as recreationally active using the IPAQ, detailed information regarding primary sport, training modality, and weekly training volume was not systematically collected. This limits the phenotypic characterization of the sample and may reduce comparability with future studies. Other variables known to influence PPT, including recent sleep quality, caffeine intake, analgesic or anti-inflammatory medication use, and menstrual cycle phase in female participants, were not systematically recorded or controlled. These factors may have contributed to inter-individual variability in baseline PPT and post-exercise PPT responses. Moreover, because PPT measurements were performed in a fixed order, with the quadriceps always assessed before the thenar eminence, a potential order effect cannot be excluded and may have influenced the distal PPT response. In addition, because BikeErg damper settings were self-selected rather than standardized across participants, differences in air resistance and cadence strategies may have influenced between-participant power outputs and should be considered when comparing these findings with studies using electromagnetically braked ergometers or standardized resistance settings. Given this was an exploratory first-step study, no comparison with other exercise modalities or intensities was included. The absence of a non-exercise or time-matched control condition prevents causal attribution to SIT alone, as repeated algometric testing, familiarization, and time-related changes may have contributed to the observed PPT increases. Therefore, the present findings cannot determine whether this brief SIT protocol is superior, equivalent, or inferior to other exercise approaches for inducing EIH. Future studies should compare this brief SIT protocol with a non-exercise control condition and with time- or volume-matched moderate-intensity continuous exercise.

## 5. Conclusions

**Figure 2:**
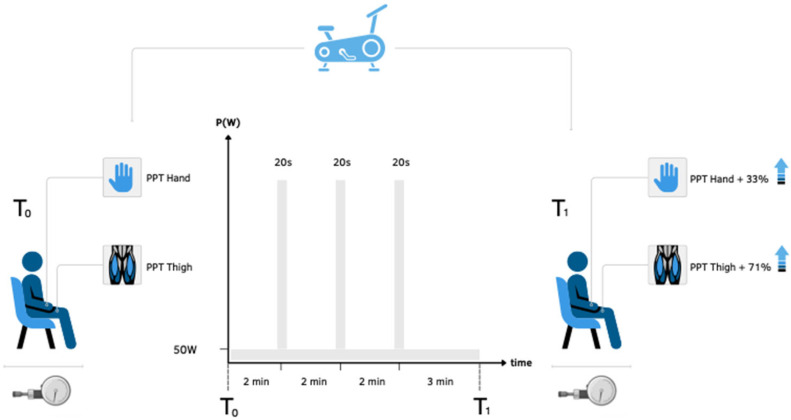
Infographic of the SIT protocol adopted and results on Pain Pressure Threshold (PPT) for the local site (PPT Thigh) and distal site (PPT Hand).

In this exploratory single-arm study, PPT increased significantly at both local and distal sites following a brief SIT session in healthy adults, with the protocol and results summarized in Figure 2. These changes were not correlated with RPE or the average power expressed during the sprints. Future controlled studies are required to compare SIT with non-exercise control conditions and other aerobic exercise modalities, including time- or volume-matched moderate-intensity continuous exercise.

## Figures and Tables

**Table 1:** Descriptive characteristics of the participants with data shown as mean ± standard deviations (SD).

Variable	Data
Female/male sex (*n*)	15/29
Age (yr)	30.5 ± 4.4
Height (m)	1.75 ± 0.1
Weight (kg)	73 ± 11.1
BMI (kg/m^2^)	23.6 ± 1.9
IPAQ (*n*) moderate/high	6/38

yr = years; m = meters; kg = kilograms; BMI = body mass index; IPAQ = International Physical Activity Questionnaire.

**Table 2:** Results presented as mean ± standard deviation (SD).

Variable	Pre	Post	% Change	ES (95% CI), *p*-Value
PPT Thigh (kgf/cm^2^)	3.83 ± 1.32	6.19 ± 1.78	70.90 ± 56.16	1.79 (1.31 to 2.27), *p* < 0.001
PPT Hand (kgf/cm^2^)	3.10 ± 1.21	3.94 ± 1.21	32.84 ± 31.75	1.16 (0.77 to 1.54), *p* < 0.001

PPT = pain pressure threshold; kgf = kilogram-force; cm^2^ = square centimeter; CI = confidence interval; ES = effect size.

**Table 3:** Pearson correlations of percentage changes in PPT Thigh and PPT Hand with average power during sprints (W) and RPE.

Variable	Statistic	Average Power (W)	RPE
% Change PPT Thigh (kgf/cm^2^)	*r*	−0.08	−0.03
	*95% CI*	−0.37 to 0.22	−0.32 to 0.27
	*p*	0.63	0.86
% Change PPT Hand (kgf/cm^2^)	*r*	−0.04	0.05
	*95% CI*	−0.33 to 0.26	−0.25 to 0.34
	*p*	0.78	0.74

PPT = Pain Pressure Threshold; kgf = kilogram-force; cm^2^ = square centimeter; % = percentage; W = watt; RPE = Rating of Perceived Exertion.

## Data Availability

The data presented in this study are available on request from the corresponding author. The data are not publicly available due to privacy and ethical restrictions.

## Abbreviations

The following abbreviations are used in this manuscript:

CI	Confidence Interval
EIH	Exercise-Induced Hypoalgesia
ES	Effect Size
PPT	Pain Pressure Threshold
RPE	Rate of Perceived Exertion
SIT	Sprint Interval Training
